# Flight performance in the altricial zebra finch: Developmental effects and reproductive consequences

**DOI:** 10.1002/ece3.2775

**Published:** 2017-03-09

**Authors:** Ondi L. Crino, Brett Klaassen van Oorschot, Kristen E. Crandell, Creagh W. Breuner, Bret W. Tobalske

**Affiliations:** ^1^ Centre for Integrative EcologyDeakin UniversityGeelongVic.Australia; ^2^ Division of Biological SciencesUniversity of MontanaMissoulaMTUSA; ^3^ Department of ZoologyUniversity of CambridgeCambridgeUK

**Keywords:** brood size, corticosterone, developmental stress, fitness, flight velocity, locomotor performance, takeoff

## Abstract

The environmental conditions animals experience during development can have sustained effects on morphology, physiology, and behavior. Exposure to elevated levels of stress hormones (glucocorticoids, GCs) during development is one such condition that can have long‐term effects on animal phenotype. Many of the phenotypic effects of GC exposure during development (developmental stress) appear negative. However, there is increasing evidence that developmental stress can induce adaptive phenotypic changes. This hypothesis can be tested by examining the effect of developmental stress on fitness‐related traits. In birds, flight performance is an ideal metric to assess the fitness consequences of developmental stress. As fledglings, mastering takeoff is crucial to avoid bodily damage and escape predation. As adults, takeoff can contribute to mating and foraging success as well as escape and, thus, can affect both reproductive success and survival. We examined the effects of developmental stress on flight performance across life‐history stages in zebra finches (*Taeniopygia guttata*). Specifically, we examined the effects of oral administration of corticosterone (CORT, the dominant avian glucocorticoid) during development on ground‐reaction forces and velocity during takeoff. Additionally, we tested for associations between flight performance and reproductive success in adult male zebra finches. Developmental stress had no effect on flight performance at all ages. In contrast, brood size (an unmanipulated variable) had sustained, negative effects on takeoff performance across life‐history stages with birds from small broods performing better than birds from large broods. Flight performance at 100 days posthatching predicted future reproductive success in males; the best fliers had significantly higher reproductive success. Our results demonstrate that some environmental factors experienced during development (e.g. clutch size) have stronger, more sustained effects than others (e.g. GC exposure). Additionally, our data provide the first link between flight performance and a direct measure of reproductive success.

## 1 Introduction

The developmental environment can have pervasive and sustained effects on animal phenotype and performance. Environmental conditions experienced during development can induce phenotypic responses that transmit information about the postnatal environment and are sustained across life‐history stages (i.e., developmental programming; Catalani et al., 2000; McMillen & Robinson, 2005). The stress hormones, glucocorticoids, are one important mediator between the developmental environment and the postnatal phenotype. Like adults, developing animals can be exposed to glucocorticoids directly when environmental stimuli activate the hypothalamic–pituitary–adrenal (HPA) axis and result in the release of endogenous glucocorticoids (e.g., Blas, Baos, Bortolotti, Marchant, & Hiraldo, 2005; Kitaysky, Kitaiskaia, Wingfield, & Piatt, 2001; Wada et al., 2008). Developing animals can also be indirectly exposed to glucocorticoids via egg yolk in viviparous animals or placental interactions and breast milk in mammals (e.g., Brummelte, Schmidt, Taves, Soma, & Galea, 2010; Hayward & Wingfield, 2004; Tarlow, Wikelski, & Anderson, 2001). Across taxonomic groups, exposure to glucocorticoids and other stressors (e.g., food restriction) during development (hereafter developmental stress) has myriad phenotypic effects including decreased growth and development, impaired immune function, elevated stress responses, altered cognitive function, and changes in reproductive success (reviewed in Henriksen, Rettenbacher, & Groothuis, 2011; Matthews, 2002; Nesan & Vijayan, 2013; Schoech, Rensel, & Heiss, 2011).

Although many of the phenotypic effects of developmental stress appear negative, developmental stress may alternatively have positive effects on phenotype (e.g., Chin et al., 2009; Crino, Driscoll, & Breuner, 2014; Crino, Driscoll, Ton, & Breuner, 2014; Crino, Prather, Driscoll, Good, & Breuner, 2014), and there is increasing evidence that developmental stress can induce adaptive phenotypic responses (e.g., Love & Williams, 2008). Developmental stress could maximize fitness by matching offspring needs to parental capabilities or by inducing phenotypic changes which prepare animals to live in harsh environments (Breuner, 2008; Coslovsky & Richner, 2011; Love & Williams, 2008). Although the phenotypic effects of developmental stress have been well described across taxonomic groups, there are comparatively few studies that have sought to quantify the fitness consequences of developmental stress (but see Crino, Prather, et al., 2014; Naguib, Nemitz, & Gil, 2006). Admittedly, this is a challenging question to address in free‐living animals, and studies with captive animals can be difficult to interpret in an ecological and evolutionary context. Given these limitations, studies that investigate the effects of developmental stress on fitness‐related traits provide insight into how the developmental environment affects fitness (e.g., Strange, Bowden, & Thompson, 2016). Here, we suggest that locomotor performance (e.g., flight performance in birds) is an ideal metric to enable testing for effects of developmental stress on fitness because it is tractable to assess in the laboratory and has clear implications for fitness (e.g., Husak, 2006; Husak, Fox, Lovern, & Van Den Bussche, 2006; Le Galliard, Clobert, & Ferriere, 2004; reviewed in Irschick, Meyers, Husak, & Le Galliard, 2008).

In volant birds, flight performance contributes to many ecologically relevant functions, including mate displays (Clark, 2009; Clark & Feo, 2008; Usherwood, 2008), prey capture (Helms, Godfrey, Ames, & Bridge, 2016; Warrick, Hedrick, Biewener, Crandell, & Tobalske, 2016), predator escapes (Kullberg, Fransson, & Jakobsson, 1996; Kullberg, Jakobsson, & Fransson, 2000; van den Hout, Mathot, Maas, & Piersma, 2010), and arrival time to migration grounds (Bowlin & Winkler, 2004; Matyjasiak, 2013). Thus, flight performance can potentially affect both reproductive success (via mate choice; reviewed in Barske, Schlinger, Wikelski, & Fusani, 2011; Byers, Hebets, & Podos, 2010) and survival (Møller, 2010). Developmental stress affects flight performance in juvenile and adult birds with evidence for either positive or negative effects. In the European starling (*Sturnus vulgaris*), developmental stress via experimentally elevated glucocorticoids *in ovo* increased initial flight performance (rate gain of kinetic and potential energy) in juvenile birds, suggesting that glucocorticoids may have a preparative role for animals developing in stressful environments (Chin et al., 2009). Conversely, other studies have found that adverse developmental conditions negatively influence flight performance. European starlings cross‐fostered to nests where they were slightly smaller than nest mates (putatively making them less competitive with nest mates for food; Cotton, Wright, & Kacelnik, 1999) faced steeper trade‐offs in flight performance parameters as adults (O'Hagan, Andrews, Bedford, Bateson, & Nettle, 2015). Similarly, nestling European starlings reared by mothers with decreased condition (via feather clipping) had decreased flight performance as fledglings (males only; Verspoor, Love, Rowland, Chin, & Williams, 2007). Cumulatively, these studies demonstrate that the developmental environment can alter flight performance ability at some life‐history stages. However, the phenotypic effects of developmental glucocorticoid exposure do not always track across life‐history stages, and effects that are present at one stage may be absent at others (e.g., Crino, Driscoll, & Breuner, 2014; Lendvai, Loiseau, Sorci, & Chastel, 2009). Therefore, studies that address the longitudinal effects of the early environment on phenotype have much to contribute to our understanding of the fitness consequences of the early developmental environment.

**Figure 1:**
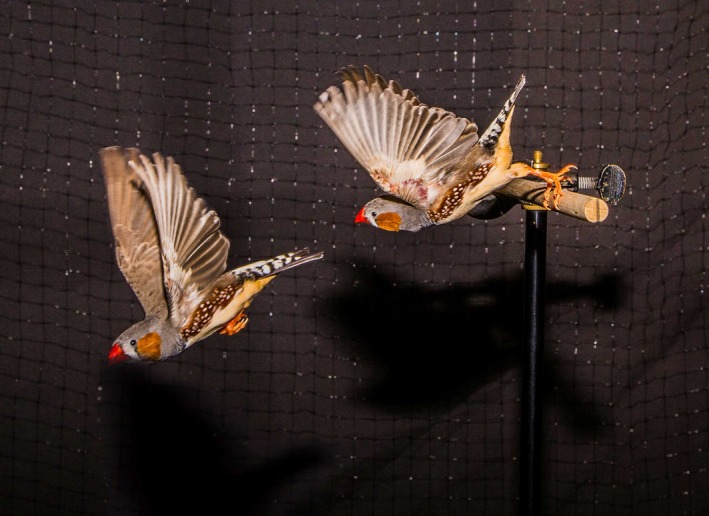
An adult zebra finch (*Taeniopygia guttata*) takes flight. Photo credit: Brett Klaassen van Oorschot and Robert Niese

The goal of this experiment was to examine the effects of the postnatal developmental environment on takeoff flight performance in male and female zebra finches (Figure 1) across life‐history stages. We examined the effects of both clutch size (an unmanipulated variable) and experimentally elevated corticosterone (CORT, the dominant avian glucocorticoid) on flight performance. We fed nestling zebra finches CORT during the nestling period (12–28 days posthatch) and compared flight performance parameters (flight velocity and ground‐reaction forces during takeoff) at 30, 60, and 100 days posthatch with those of birds fed a control treatment (vehicle alone) during the nestling period. In a subset of adult males, we tested for associations between flight performance and reproductive success (derived from a series of common garden breeding experiments from a separate study). We predicted that experimental treatment with CORT during development would have positive effects on flight performance and that these effects would be sustained across life‐history stages. Additionally, we predicted that flight performance would be positively associated with reproductive success in male zebra finches. These experiments uniquely allow us to examine the effects of developmental stress on flight performance across life‐history stages, and to tie those effects directly to reproductive success.

## 2 Materials and Methods

### 2.1 Parental birds—housing and breeding

The breeding colony consisted of ten female and ten male zebra finches (*Taeniopygia guttata*) that were sourced from pet stores. Throughout the course of the experiment, we replaced three males and two females that died. Breeding finches were housed in a 3.5 × 3.5 × 3.5 m room where they were allowed to interact freely with all other birds. We housed the birds on a 14:10 light/dark cycle at 26–27°C with 20%–30% humidity. Birds had access to 12 nest boxes and shredded burlap nesting material. We fed birds commercial finch seed (Silver Song West) and spray millet ad libitum and supplemented their diet daily with hard boiled eggs, spinach, and crushed egg shells. We monitored the nest boxes daily for signs of breeding activity.

### 2.2 Nestlings—treatment and measurements

Nestlings were randomly assigned to treatment groups (CORT or control). Nestlings exposed to the CORT treatment were fed oral boluses (25 μl) of CORT (Sigma‐Aldrich) dissolved in peanut oil twice daily approximately 5 hrs ±1 hr apart. From 12 to 15 days posthatch, nestlings received 0.124 mg/ml of CORT in peanut oil for a total daily dose of 6.2 μg of CORT. Starting 16 days posthatch, the dose was increased to 0.163 mg/ml for a total daily exposure of 8.15 μg of CORT. Control nestlings were fed 25 μl of peanut oil on an identical feeding schedule. Nestlings were exposed to treatments from 12 to 28 days posthatch (methods as per Crino, Driscoll, & Breuner, 2014; Spencer, Evans, & Monaghan, 2009). Following nutritional independence (30 days posthatching), we removed the parents and supplied the nestlings with ad libitum commercial finch food, spray millet, boiled eggs, spinach, and crushed egg shells.

We measured tarsus, “museum” wing chord (i.e., with wing folded, distance from wrist to the distal tip of the primaries), and mass at 30, 60, and 100 days posthatching. We calculated condition for birds at all ages using the scaled mass index (Peig & Green, 2009). The scaled mass index accounts for errors associated with residual body mass measurements using a scaling relationship derived from a standardized major axis regression of mass on a linear body measurement (wing chord in our experiment) to calculate the expected mass of each individual at a fixed body size (Peig & Green, 2009). Expected mass is calculated using the regression equation. In this way, the scaled mass index standardizes all animals to the same growth phase or body size and is considered to be a more accurate measure of condition (Peig & Green, 2010). This index has been used to calculate condition across taxonomic groups (e.g., Cox & Calsbeek, 2015; Frincke‐Craig et al., 2015; Kelly, Tawes, & Worthington, 2014). Scaled mass is hereafter referred to as “condition” and presented in units of grams.

Birds were repeatedly sampled for flight performance at 30, 60, and 100 days posthatching except when they died or were being used in other experiments. Our sample sizes were as follows: 30 days posthatching *n* = 13 for control and 16 for CORT‐fed birds; 60 days posthatching *n* = 7 for control and 15 for CORT‐fed birds; and 100 days posthatching *n* = 6 for control and 12 for CORT‐fed birds. For broods of 4, 5, and 6, our sample sizes for brood size analyses were as follows: 30 days posthatching *n* = 8, 10, and 11; 60 days posthatching *n* = 3, 8, and 11; 100 days posthatching *n* = 2, 7, and 9.

### 2.3 Flight kinematics and ground‐reaction forces

We recorded the flight trials using a Photron Fastcam‐1024PCI (Photron USA, Inc. San Diego, CA, USA) recording at 500 Hz with a shutter speed of 150–200 μs. The camera was oriented orthogonally to the birds’ flight trajectory and captured their takeoff during ground contact and for the first ~10 wingbeats of flight. We placed small adhesive markers on their chest and hip to assist in digitization. Birds were digitized using the DLTdv5 script (Theriault, Fuller, et al. 2014) in MATLAB (R2011b; Mathworks Inc., Natick, MA, USA). All flight variables were calculated from one flight that was initiated by slowly approaching the bird with a novel object. To determine flight velocity, a marker at the belly was used as an approximation of the center of mass. Flight velocity was averaged for the first three wingbeats following the end of force application to the perch.

To measure the contribution of leg forces during takeoff, the birds started their flight from a perch 1.3 cm in diameter affixed to a custom force plate (15 × 15 cm platform, 200 Hz resonating frequency; Bertec Corporation, Columbus, OH, USA). Forces were digitally amplified 5× (adult birds) and 10× (juvenile birds) using a Bertec model M6810 amplifier. We then recorded the data at 1000 Hz using an ADInstruments PowerLab and Chart v5.2 software (ADInstruments, Colorado Spring, CO, USA). Forces were filtered using a zero phase shift low‐pass (50 Hz) Butterworth filter. Resultant (i.e., summed vertical and horizontal) forces during takeoff are hereafter referred to as ground‐reaction forces.

To measure relative hind limb contribution to initial resultant velocity (hereafter takeoff velocity), we followed the methods of Earls (2000), Tobalske, Altshuler, and Powers (2004) and Provini, Tobalske, Crandell, and Abourachid (2012, 2014) by integrating velocity with respect to time from the beginning of takeoff to the foot leaving the perch. Peak ground‐reaction force was measured as the resultant of the maximum force applied in the vertical direction and the force applied at the horizontal direction during maximum vertical force application. Impulse was calculated as the integral of ground‐reaction forces with respect to time and represents the hind limb contribution to initial takeoff velocity.

Overall, these kinematic and ground force calculations resulted in four variables that we used to describe flight performance: flight velocity, takeoff velocity, peak ground‐reaction force, and impulse. Flight velocity is the speed of flight birds achieved by the end of the third wingbeat following toe‐off. Takeoff velocity describes the speed of the birds at toe‐off. Ground‐reaction forces represent the contribution of the legs to takeoff velocity. Impulse is an additional measure of change in momentum that incorporates the time course of force application to the perch (i.e., impulse = force × time; takeoff velocity = impulse/mass).

### 2.4 Reproductive success

We quantified reproductive success in a separate experiment that examined the effects of developmental CORT exposure on alternative reproductive tactics in male zebra finches (Crino, Prather, et al., 2014). For this experiment, we conducted a series of four common garden breeding experiments that consisted of five control males, five CORT‐fed males, and 10 unmanipulated females that were allowed to breed for one reproductive bout. As part of this experiment, we measured the number of nestlings reared with a social mate (social paternity) and the number of nestlings sired with a social mate and through extrapair copulations (EPCs) with other females (genetic paternity). Comprehensive details of the methods used to determine social and genetic paternity are found in Crino, Prather, et al. (2014). We compared flight performance metrics in males at 100 days posthatching (the time point closest to reproductive maturity) to reproductive success data. The comparison of reproductive success and flight performance data is a post hoc analysis on a subset of males used in both experiments (*n* = 7).

### 2.5 Statistical analyses

All morphological and condition data (the scaled mass index) were normally distributed (Shapiro–Wilk, *p* > .10). We used multivariate general linear models to examine the effects of treatment, sex, and brood size on morphology and condition. Sex had no significant effects on body size or condition and was removed from the final model. We used least significant difference post hoc analyses to test for significant difference between body measurements and brood size.

Departures from normality for all flight performance parameters were small (Shapiro–Wilk, *p* > .24). We used separate linear mixed‐effects models to examine the effects of CORT treatment on flight performance parameters (takeoff velocity, ground‐reaction forces, flight velocity, and impulse). In each model, we used bird identity and nest identity as subject variables to control for repeated measurements from individuals and for nonindependence of nestlings from the same natal nest (respectively). We used treatment, sex, brood size, age (30, 60, or 100 days), and treatment × age (to examine the effect of treatment at each age class) as fixed factors. Brood size affected flight performance parameters. We investigated significant differences between brood sizes at different ages using multivariate general linear models and least significant difference post hoc analyses. Sample sizes varied between age groups because of natural death and logistical constraints on the colony size in the housing facility. Sample sizes for takeoff velocity, ground‐reaction forces, and impulse for control and CORT‐fed treatment groups were as follows: *n* = 13, 16 at 30 days posthatching; 7, 15 at 60 days posthatching; and 6, 12 at 100 days posthatching. We were unable to obtain flight velocity data from some of the videos collected if the focal bird did not fly in a linear path. For this reason, sample sizes for analyses involving flight velocities for control and CORT‐fed birds were *n* = 7, 12 at 30 and 60 days posthatching and *n* = 6, 12 at 100 days posthatching. We used multivariate linear regressions with backward elimination to examine associations between flight performance metrics and social and genetic paternity. Unless indicated, figures present data pooled from males and females.

## 3 Results

### 3.1 Body size—treatment and effects of brood size

#### 3.1.1 Treatment effects

**Figure 2:**
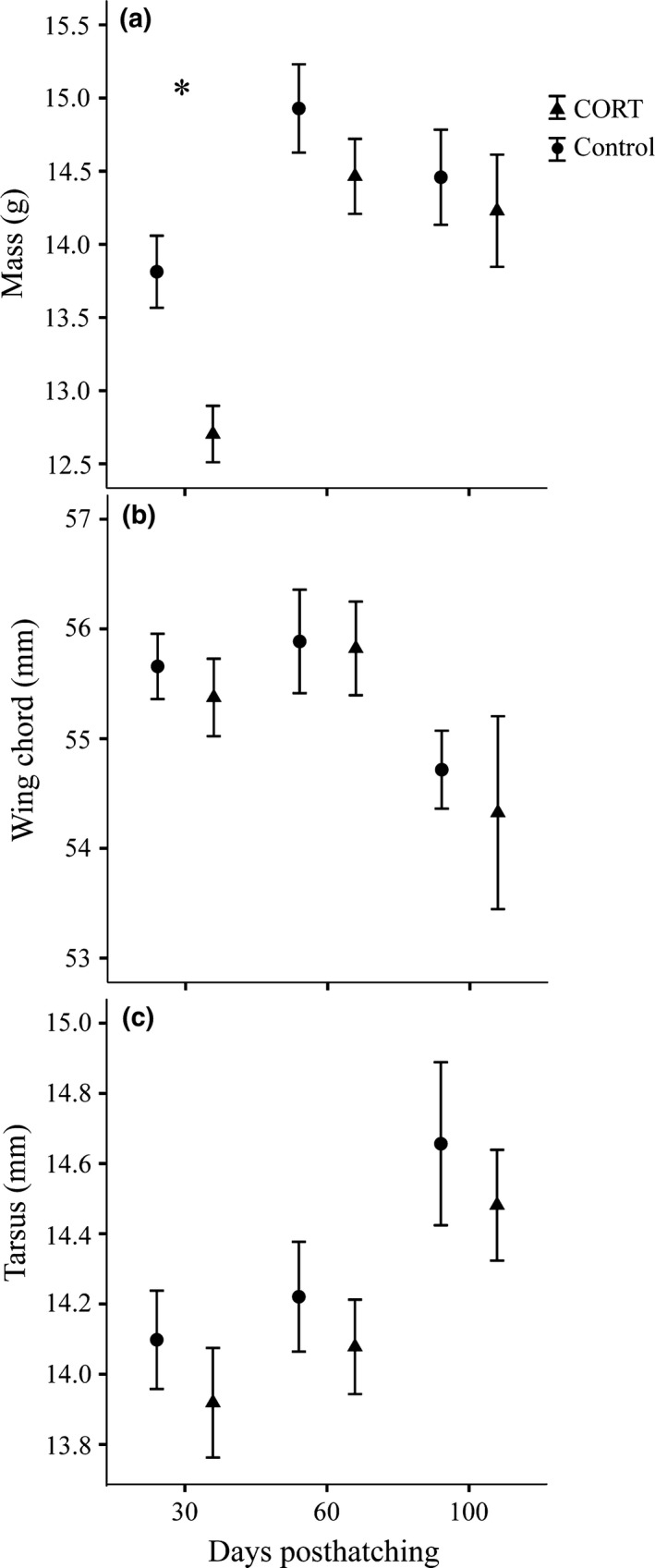
The effects of CORT treatment on body size at 30, 60, and 100 days posthatching for (a) body mass, (b) wing chord, and (c) tarsus. Bars represent ±1 *SEM*

At 30 days posthatching, nestlings fed CORT had lower masses (*F*_1,28_ = 13.50, *p* = .001) and were in poorer condition (*F*_1,28_ = 5.14, *p* = .03) compared to control nestlings. There were no differences between treatment groups in tarsus (*F*_1,28_ = 0.76, *p* = .40) or wing chord (*F*_1,28_ = 0.20, *p* = .66) at 30 days posthatching. At both 60 and 100 days posthatching, there were no differences between treatment groups in tarsus, wing chord, mass, or condition (*F* < 1.72, *p* > .21, *F* < 0.80 for all; Figure 2).

#### 3.1.2 Effects of brood size

**Figure 3:**
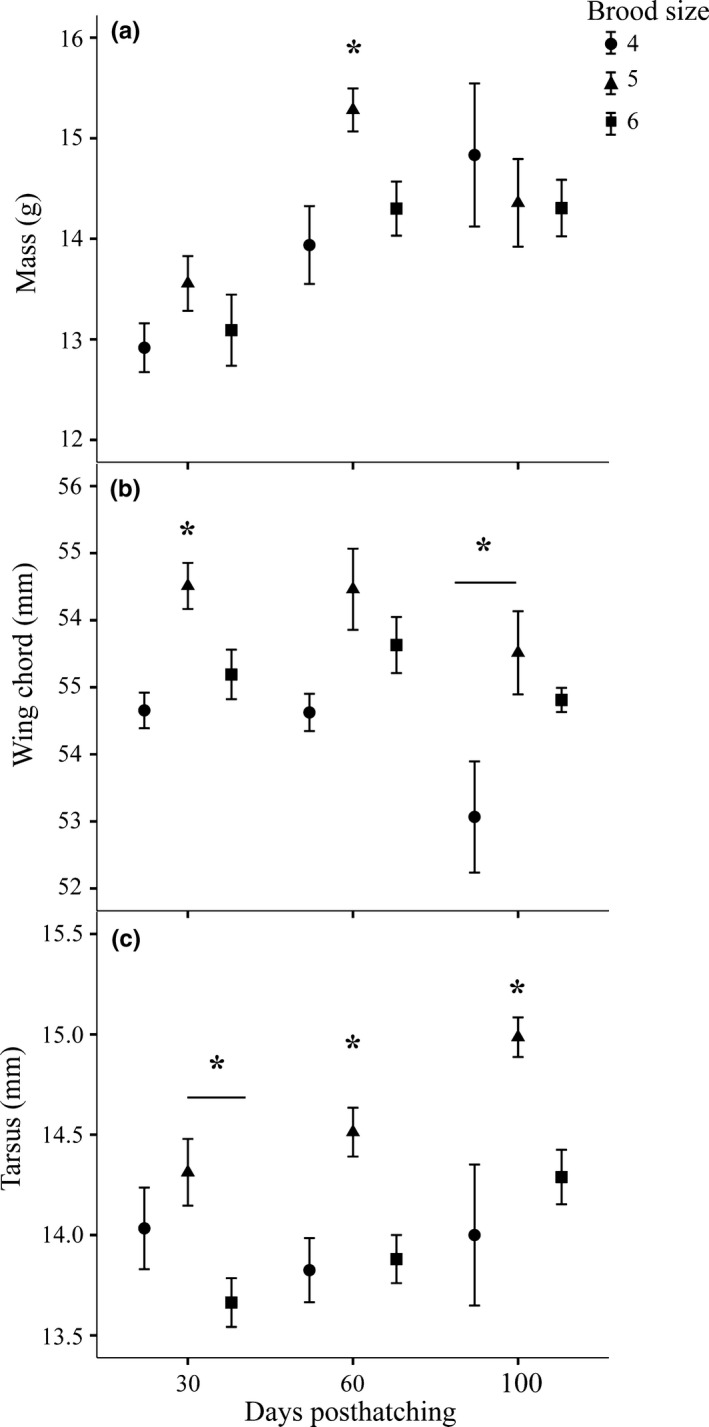
The effects of brood size on body size at 30, 60, and 100 days posthatching for (a) body mass, (b) wing chord, and (c) tarsus. Bars represent ±1 *SEM*. *p < .05

Brood size affected body size in birds at 30, 60, and 100 days posthatching (Figure 3). Specifically, at 30 days posthatching, birds from broods of 5 had longer tarsi compared to birds from broods of 6 (*p* = .01) and longer wing chords than birds from broods of 4 and 6 (*p* = .001, .01, respectively). At 60 days posthatching, birds from broods of 5 had longer tarsi than birds from broods of 4 and 6 (*p* = .007, .002, respectively) and were heavier than birds from broods of 4 and 6 (*p* = .001, .01). At 100 days posthatching, birds from broods of 5 had longer tarsi compared to birds from broods of 4 and 6 (*p* = .02, .004, respectively) and birds from broods of 4 had shorter wing chords compared to birds from broods of 5 and 6 (*p* = .002, .009, respectively). These data are congruous with data from a larger sample size from this population of zebra finches showing that birds reared in medium‐sized broods are larger than birds reared in small and large broods for the duration of their lives (Crino, Driscoll, & Breuner, 2014).

### 3.2 Flight performance—treatment and brood size effects

**Figure 4:**
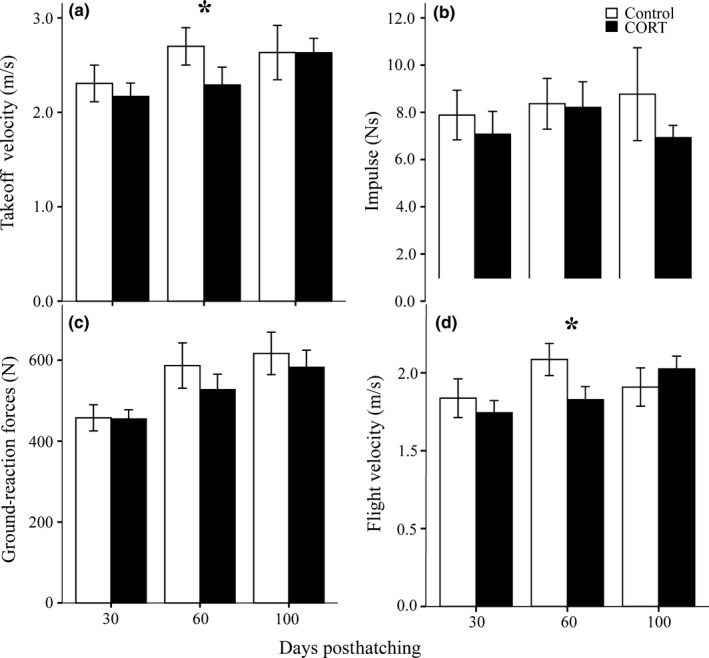
Mean flight performance responses ±1 *SEM* for control (white bars) and CORT‐fed (black bars) at 30, 60, and 100 days posthatching for (a) takeoff velocity, (b) impulse, (c) peak ground‐reaction forces, and (d) flight velocity. *p < .04

There were no differences in flight performance parameters between control and CORT‐fed birds (*p* > .14 for all). Age had a significant effect on takeoff velocity (*F*_2,59_ = 3.78, *p* = .029) and ground‐reaction forces (*F*_2,59_ = 8.56, *p* = .001) with birds 30 days posthatching having the lowest values for these parameters compared to birds 60 (*p* = .052, .004, respectively) and 100 days posthatching (*p* = .013, .001, respectively). Age had no significant effect on flight velocity (*F*_2,48_ = 2.33, *p* = .108) or impulse (*F*_2,59_ = 0.574, *p* = .566). There were no significant treatment × age effects for takeoff velocity (*F*_2,59_ = 0.992, *p* = .377), ground‐reaction forces (*F*_2,59_ = 0.499, *p* = .610), flight velocity (*F*_1,48_ = 1.297, *p* = .283), or impulse (*F*_2,59_ = 0.226, *p* = .798; Figure 4). Sex had no effect on takeoff velocity (*F*_2,59_ = 0.089, *p* = .915), ground‐reaction forces (*F*_2,59_ = 0.230, *p* = .796), flight velocity (*F*_1,48_ = 0.003, *p* = .956), and impulse (*F*_2,59_ = 0.267, *p* = .767).

**Figure 5:**
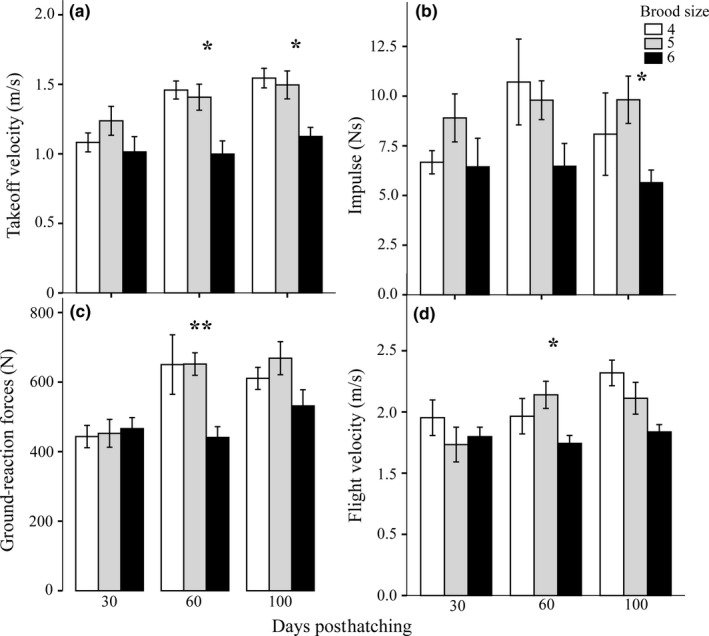
Mean flight performance responses ±1 *SEM* for zebra finches from broods of four (white bars), five (gray bars), and six (black bars) at 30, 60, and 100 days posthatching for (a) takeoff velocity, (b) impulse, (c) peak ground‐reaction forces, and (d) flight velocity. **p < .001, *p < .05

Brood size had strong effects on takeoff velocity (*F*_2,59_ = 9.94, *p* < .0001), ground‐reaction forces (*F*_2,59_ = 5.802, *p* = .005), flight velocity (*F*_2,48_ = 4.64, *p* = .014), and impulse (*F*_2,59_ = 5.510, *p* = .006), but these effects were highly dependent on the age of the birds. At 30 days posthatching, there were no differences in any flight performance parameter between birds from clutches of 4, 5, or 6 (*F*_2_ < 0.65, *p* > .53 for all). For birds 60 days posthatching, brood size had strong effects on ground‐reaction forces (*F*_2_ = 12.57, *p* = .001, Figure 5c), flight velocity (*F*_2_ = 5.12, *p* = .02, Figure 5d), and takeoff velocity (*F*_2_ = 5.57, *p* = .02, Figure 5a). There was a nonsignificant trend for brood size to affect impulse (*F*_2_ = 3.09, *p* = .08, Figure 5b). Birds from broods of 6 produced lower ground‐reaction forces during takeoff compared to birds from broods of 4 or 5 (*p* = .03, .001), had lower flight velocities than birds from broods of 5 (*p* = .006), and had lower takeoff velocities than birds from broods of 4 or 5 (*p* = .05, .008). At 100 days posthatching, brood size also affected takeoff velocity and impulse (*F*_2,2_ = 6.66, 5.33, *p* = .08, .02, respectively). Similar to the results of birds at 60 days posthatching, birds from broods of 6 had lower takeoff velocities compared to birds from broods of 4 and 5 (*p* = .03, .005, respectively). Birds from broods of 6 also had lower impulse compared to birds from broods of 5 (*p* = .005). There was a nonsignificant trend for clutch size to affect flight velocity (*F*_2_ = 3.22, *p* = .07) and no effect of brood size on ground‐reaction forces (*F*_2_ = 2.23, *p* = .14).

In summary, developmental CORT treatment had short‐term effects on body size and no measurable effects on flight performance. In contrast, brood size had more sustained effects on flight velocity for birds at 60 and 100 days posthatching with birds from smaller broods performing better than birds from large broods.

### 3.3 Reproductive success and flight performance

**Figure 6:**
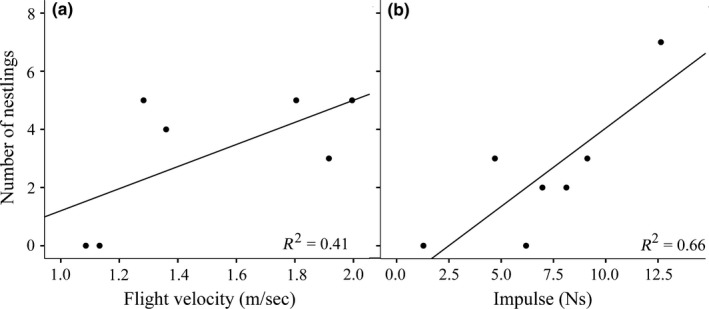
Flight performance (velocity and impulse) at 100 days posthatching and reproductive success (number of nestlings) for male zebra finches for (a) social and (b) genetic paternity

In adult males (100 days posthatching), flight performance was positively related to reproductive success. Specifically, social paternity was elevated in males with higher ground‐reaction forces (*t*_6_ = −3.35, *p *= .04) and with faster takeoff (*t*_6_ = −4.60, *p* = .02) and flight velocities (*t*_6_ = 5.42, *p* = .01, Figure 6a). Similarly, genetic paternity was elevated in males with higher impulse values compared to slower males (*t*_6_ = 2.85, *p* = .046, Figure 6b). There was a nonsignificant trend for males with faster flight velocities to sire more genetic offspring compared to slow males (*t*_6_ = 2.47, *p* = .069).

## 4 Discussion

Exposure to developmental stress has been associated with either positive or negative effects on flight performance (Chin et al., 2009; O'Hagan et al., 2015; Verspoor et al., 2007). Unlike past studies, we found that developmental CORT exposure had no effects on takeoff flight performance despite CORT‐fed birds being smaller than control birds at 30 days posthatching. We found that developmental CORT treatment had negative effects on body size, but these effects were only present in birds at 30 days posthatching. In contrast, we found that brood size had stronger and more pervasive effects on body size and flight performance across life‐history stages such that birds from large broods were smaller and performed poorly compared to birds from smaller broods (Figures 3 and 4b). In males, flight performance metrics were positively correlated with reproductive success (social and genetic paternity) such that males that were the best at taking to flight had the highest reproductive success. These results demonstrate that the early developmental environment can have sustained negative effects on flight performance. Additionally, these data link performance to reproductive success, suggesting that flight performance could have important effects on fitness (Figure 6).

Why did we observe no effect of CORT on flight performance while other studies have reported significant positive or negative effects? In general, the effects of developmental stress likely depend on the context within which the effect is measured, the type of developmental stress (e.g., food restriction versus elevated glucocorticoids), and the timing of the stressor (pre‐ or postnatal; reviewed in Crino & Breuner, 2015). Chin et al. (2009) manipulated developmental CORT by injecting CORT into eggs (i.e., prenatal manipulations), whereas we fed nestlings CORT during the postnatal period. Both of these methods have been used extensively in studies that seek to manipulate developmental CORT but can result in different phenotypic outcomes (Crino & Breuner, 2015). For example, in European starlings, prenatal stress decreases stress reactivity, while postnatal stress increases it (Love & Williams, 2008). Ecologically, prenatal CORT exposure simulates an environment where the mother has high levels of endogenous CORT at the time of egg laying (Hayward & Wingfield, 2004; Saino et al., 2005). In contrast, elevated postnatal CORT simulates an environment where nestlings experience stress in response to sibling competition, nutritional deprivation, inclement weather, predation pressure, or anthropogenic disturbance. In some scenarios, the environmental factors that contribute to elevated maternal and nestling CORT may be similar in origin (e.g., inclement weather could result in elevated CORT in adults and nestlings). In this way, CORT exposure during the pre‐ and postnatal periods could be seen as providing the same cue to developing birds (i.e., the postnatal environment is stressful). However, pre‐ and postnatal stress exposure can produce opposite phenotypic responses (e.g., Boogert, Zimmer, & Spencer, 2013; Homberger, Jenni‐Eiermann, Roulin, & Jenni, 2013; Lehmann, Stohr, & Feldon, 2000; Vallee et al., 1997), suggesting that the timing of perinatal stress is relevant to how it affects developing animals (discussed in O'Hagan et al., 2015; Strange et al., 2016). An alternative, but not mutually exclusive, hypothesis is that prenatal CORT exposure alters nestling phenotype in ways that benefit the mother (e.g., smaller nestlings; Love & Williams, 2008), whereas postnatal CORT affects nestling morphology and physiology in order to increase survival for the nestling. Evaluating these hypotheses in terms of the effects of developmental CORT exposure on flight performance would help illuminate the functional differences between pre‐ and postnatal CORT exposure in an ecologically relevant trait.

In birds, the hind limbs contribute virtually all of the initial acceleration of takeoff until the feet leave the terrestrial substrate and then wings and forelimbs generate most of the lift for flight (Earls, 2000; Provini et al., 2012). Thus, measuring ground‐reaction forces as well as flight velocity provides a novel understanding of how the developmental environment affects two distinct components of takeoff performance. Although takeoff speed is clearly important for escaping predators, the speed following takeoff is likely to be important as well. The additional variables we measured add valuable insight into how early developmental condition affects different morphological modules associated with flight performance (i.e., legs versus wings; Gatesy & Dial, 1996; Heers & Dial, 2015).

We found brood size had strong negative effects on peak ground‐reaction forces and impulse during takeoff and flight velocities immediately following takeoff at 60 and 100 days posthatching. Flight velocity, peak ground‐reaction force, and impulse during takeoff are all likely correlated with body size of the bird. Birds from small and large broods had the smallest body sizes across time, so we suggest that body size may be the most robust descriptive factor for takeoff and flight performance. In animal flight, the work and power required to move through the air scale approximately with body mass, but the available power for flight does not increase at the same rate such that mass‐specific performance declines with body size (Ellington, 1991; Hill, 1950; Jackson & Dial, 2011). Increased payload (i.e., nonmuscle additions such as seeds, fat, or eggs) increases the work and power requirement and thus affects performance. For example, in great tits, females have lower initial takeoff flight velocities after a day of foraging compared to preforaging dawn flights (Krams, 2002).

Birds face trade‐offs in locomotor development as they transition from nestlings to adults. For example, allocation of resources to forelimb development (i.e., wings) can have adverse effects on hind limb locomotor performance. Developmental stress may influence the ontogenetic trajectory of birds by shifting resources to different locomotor units. Furthermore, mass may be particularly significant for flight performance in developing birds that must first reach a certain level of wing development and then drop below a critical weight to wing size ratio in order to fly and reduce mass‐dependent flight costs (Sprague & Breuner, 2010; Witter & Cuthill, 1993). Support for this is found in Laysan albatross chicks (*Phoebastria immutabilis*) that lose body mass prior to fledging and delay fledging when experimentally supplemented with food (resulting in chicks that were too heavy to fly; Sprague & Breuner, 2010). Similarly, in common swifts (*Apus apus*), nestlings facultatively adjust their body mass in order to optimize aerodynamic performance at fledging (Wright, Markman, & Denney, 2006).

Morphological dimensions may contribute differently to performance in juveniles compared with adults. New research reveals that takeoff performance in adult tits decreases as wing loading (weight per unit wing area) increases (McFarlane, Altringham, & Askew, 2016). Although we did not measure wing area, our measure of wing chord indicates that birds from broods with four nestlings had smaller wings given their body mass (Figure 3), and it is likely they had higher wing loading. In contrast to the pattern in adult tits, these juveniles performed better than juveniles from broods of six where wing chord was larger. Perhaps these differences are because other tissues, including the neuromuscular system, are developing at the same time as the external dimensions in juveniles; this idea merits further study.

In our experiment, brood size may have contributed to variation in body size (and thus flight performance) by influencing the amount of food nestlings receive from parents. An optimal brood size may exist that minimizes the feeding costs per nestling (Ydenberg, 1994). Once parents have exerted the energy to forage for food and provision nestlings, up to a point, each additional nestling in a brood represents a smaller energetic cost to the parent. In this way, broods smaller than the optimum could have higher feeding costs per nestlings and thus do not maximize reproductive output per energy expenditure resulting in lower previsioning rates and smaller nestlings. At larger brood sizes (5+), parents may be provisioning at their maximum ability, but may simply not be able to provide sufficient food for such a large number of nestlings (Guindre‐Parker et al., 2013; Williams & Fowler, 2015). In this way, we suggest that small and large brood sizes could mimic nutritional stress by influencing the amount of food provisioned to developing nestlings. In this experiment, we see evidence to support this hypothesis with birds from broods of 5 being generally larger than birds from broods of 4 or 6 (Figure 3). These results are similar to those in a past study with a larger sample of zebra finches from the same population of zebra finches (Crino, Driscoll, & Breuner, 2014). If variation in brood size does mimic nutritional stress, it suggests that nutritional stress has more sustained effects on morphology and performance than CORT exposure. This is supported by a study by Criscuolo et al. (2011) who found that restricted nutrition during development had negative effects on vertical flight in adult female zebra finches.

Finally, we found that males that were the best at takeoff flight reared the most offspring through a social partner and sired the most offspring with their social mate and through EPCs. These data directly tie whole‐organism locomotor performance to fitness as measured through reproductive success. Locomotor performance integrates morphological, physiological, and biochemical traits into an ecologically relevant measure that has clear implications for fitness through both survival and reproductive success (Arnold, 1983; Irschick & Garland, 2001). The majority of studies relating locomotor performance to fitness focus on its effects on survival (reviewed in Irschick & Garland, 2001). However, a few studies have found positive relationships between performance and indirect measures of reproductive success (e.g., Bowlin & Winkler, 2004; Lappin & Husak, 2005), and one study has directly linked locomotor performance to reproductive success in collared lizards (*Crotaphytus collaris*; Husak et al., 2006). As far as we know, this is the first study to directly link flight performance in birds to reproductive success. Flight performance in zebra finches could be related to overall quality, and, therefore, males that are good fliers may have other high‐quality traits that contribute to increased reproductive success. Alternatively, sexual selection may be directly acting on flight performance and its underlying morphological and physiological traits if females use flight performance to evaluate males as potential mates or for EPCs. The sample size for these analyses is admittedly small (*n* = 7). Nonetheless, our data provide the first support to link flight performance to reproductive success in birds and support the use of flight performance as an ecologically and evolutionally relevant metric with which to evaluate the effects of the developmental environment on fitness.

In a previous study, we showed that the development CORT treatment described here had positive effects on male reproductive success (Crino, Prather, et al., 2014). Here, however, we show no effect of developmental CORT treatment on flight performance despite finding a link between flight performance and reproductive success. This discrepancy may be due to the fact that we examined correlations between flight performance and reproductive success and that even within treatment groups (CORT‐fed and control), there is variation in flight performance. Additionally, developmental stress has system‐wide effects on morphology, physiology, and behavior (reviewed in Crino & Breuner, 2015; Grindstaff, 2016; Schoech et al., 2011), and it is very likely that our developmental CORT treatment had phenotypic effects beyond what we quantified in the experiment described here or in previous studies (e.g., Crino, Driscoll, & Breuner, 2014; Crino, Driscoll, & Ton, 2014; Crino, Prather, et al., 2014). From our available data, it is not possible to tease apart the relationship between developmental CORT treatment, flight performance, and reproductive success, but we suggest that future studies that widely explore the effects of developmental stress on both the physiological and motor components of flight performance may illuminate the nature of these relationships.

An increasing number of studies seek to evaluate the effects of developmental CORT exposure on ecologically relevant traits in order to understand the fitness effects of stressful natal environments. Our results demonstrate that the postnatal developmental environment can have sustained effects on takeoff flight performance and that adult flight performance is associated with future reproductive success in males. Contrary to a previous study, we found that developmental CORT treatment had no effects on flight performance. In contrast to the null effects of treatment on flight performance, we found that brood size had more sustained and pervasive effects with birds from small broods performing better than birds from large broods. Our results demonstrate that some forms of developmental stress can have stronger effects on performance traits than others and support the importance of examining developmentally induced phenotypic changes across life‐history stages.

## Conflict of Interest

The authors declare no conflict of interest.
